# Phytochemical Transformations of *Panax notoginseng* Saponins During Thermal Processing and Their Potential Implications for Iron Homeostasis Through Hepcidin-Related Pathways

**DOI:** 10.3390/molecules31213937

**Published:** 2026-11-01

**Authors:** Wei Yan, Weiyou Xie, Ruolan Bai, Ting Wang, Bo Hou, Zhaojie Wang, Rongping Zhang

**Affiliations:** 1 First Clinical Medical College, Yunnan University of Chinese Medicine, Kunming 650500, China; tcmdryan@163.com (W.Y.); xieweiyou@ynnu.edu.cn (W.X.); 2 School of Chinese Materia Medica and Yunnan Key Laboratory of Southern Medicine Utilization, Yunnan University of Chinese Medicine, Kunming 650500, China; wangt662@hotmail.com (T.W.); kib_drug@163.com (B.H.); 3 Yunnan Key Laboratory of Modern Separation Analysis and Substance Transformation, College of Chemistry and Chemical Engineering, Yunnan Normal University, Kunming 650500, China; 4 School of Life Sciences, Yunnan Normal University, Kunming 650500, China; 200072@ynnu.edu.cn; 5 Engineering Research Center for Valorization of Unique Bio-Resources in Yunnan, Ministry of Education, Kunming 650500, China

**Keywords:** *Panax notoginseng*, thermal processing, rare ginsenosides, hepcidin, inflammation, gut–liver axis, iron homeostasis

## Abstract

*Panax notoginseng* has been used in Chinese materia medica for centuries, with the classical principle “sheng da shu bu” distinguishing raw roots used for acute hemostasis from thermally processed roots traditionally used for blood tonification. However, direct experimental evidence linking processing-induced phytochemical changes to hepcidin regulation remains lacking. This review examines how thermal processing of *P. notoginseng* may influence hepcidin-related pathways by integrating evidence from phytochemical transformation, hepatocyte signaling, and gut–liver axis biology. Thermal processing induces deglycosylation, dehydration, and isomerization of native saponins, generating rare, more lipophilic ginsenosides, including 20(S)-Rg3, 20(R)-Rg3, Rk1, and Rg5. These products may have altered pharmacokinetic properties relative to their native precursors. Studies of isolated ginsenosides enriched during thermal processing in non-iron models provide indirect evidence for effects on upstream inflammatory or gut–liver pathways relevant to hepcidin induction. Microbial biotransformation of orally administered saponins may also modify intestinal barrier function and inflammatory context, but it is distinct from thermal processing. Whether a chemically characterized processed *P. notoginseng* preparation reduces hepatic *HAMP* expression or circulating hepcidin, preserves ferroportin abundance, or improves iron availability in vivo has not been established.

## 1. Introduction

*Panax notoginseng* (Burkill) F.H. Chen, commonly known as Sanqi, has been documented in Chinese materia medica for centuries. This species is widely used to manage hematological and cardiovascular disorders for its dual traditional actions of promoting hemostasis and resolving blood stasis [1,2]. The medicinally active roots and rhizomes of *P. notoginseng* contain complex secondary metabolite profiles, dominated by dammarane-type tetracyclic triterpenoid saponins [3]. Structurally divided into protopanaxadiol (PPD) and protopanaxatriol (PPT) groups, these saponins are the primary active constituents that underlie the species’ multifaceted pharmacological effects [4]. The clinical application of *P. notoginseng* follows the Traditional Chinese Medicine (TCM) principle of “*sheng da shu bu*”: raw roots for staunching hemorrhage, processed roots for tonification [5]. Raw *P. notoginseng* (RPN) is traditionally used for acute traumatic conditions to resolve blood stasis and alleviate pain. In contrast, processed *P. notoginseng* (PPN), prepared by steaming, roasting, or dry-frying, is used for chronic conditions associated with blood deficiency, fatigue, and anemia [6].

Thermal processing causes marked phytochemical transformations, including deglycosylation, dehydration, and isomerization of native polar saponins. These reactions yield rare lipophilic ginsenosides, including PPD-type derivatives (e.g., 20(*S/R*)-Rg3, Rg5, and Rk1) and PPT-type derivatives (e.g., Rh4 and Rk3), with pharmacological profiles distinct from their hydrophilic precursors [7,8,9]. Steaming roots at 120 °C for 4–6 h enriches these transformed secondary metabolites and alters antioxidant and other biological activities in experimental models [10]. Pharmacokinetic findings corroborate these structural alterations: thermal processing markedly reduces the systemic exposure (area under the concentration–time curve, AUC) of major native saponins (Rg1, Rb1, Rd, and notoginsenoside R1), whereas transformed rare ginsenosides (Rg5 and Rk1) show increased systemic exposure following administration of steamed preparations [11].

Beyond direct absorption, the bioconversion of *P. notoginseng* saponins depends substantially on gut microbiota composition and enzymatic capacity. Unabsorbed native saponins undergo microbial deglycosylation in the lower gastrointestinal tract, generating bioactive metabolites including Compound K (CK) and rare ginsenosides [12,13]. Microbial biotransformation can alter the pharmacokinetic and pharmacodynamic properties of *P. notoginseng* preparations, although the effects depend on the processing context, microbial system, and metabolites formed [14]. Bacterial β-glucosidases catalyze this deglycosylation by hydrolyzing glycosidic bonds and converting major saponins (e.g., Rb1) into less polar metabolites [15]. This microbiota-dependent biotransformation shares some deglycosylation outcomes with external thermal processing but is not biochemically equivalent to steaming [16].

The therapeutic relevance of these bioavailable, microbiota-transformed ginsenosides extends to systemic iron regulation. Systemic iron homeostasis is tightly governed by the liver-derived peptide hormone hepcidin [17]. By binding ferroportin and triggering its degradation, hepcidin restricts cellular iron export, thereby modulating intestinal iron absorption and macrophage-mediated iron recycling to regulate systemic iron availability for erythropoiesis [18,19]. Under chronic inflammation, elevated IL-6 activates hepatic JAK2/STAT3 signaling, upregulating hepcidin and restricting iron release and thereby causing persistent inflammation-driven iron sequestration that underlies anemia of inflammation (AI) [20,21,22]. Studies of isolated ginsenosides that can be enriched during thermal processing have reported modulation of inflammatory signaling, including JAK2/STAT3- and NF-κB-associated pathways, in non-iron models [23,24]. Microbiota-related changes in saponin disposition and intestinal barrier function may also limit LPS-driven inflammatory input relevant to hepcidin induction [25,26,27]. Current management of anemia of inflammation remains challenging because treatment responses, safety considerations, and the contribution of underlying disease vary across clinical settings [28].

Previous reviews have provided broad accounts of processing-driven chemical transformations and pharmacological shifts in *P. notoginseng* [5]. Building on this literature, the present review distinguishes four evidence streams: native saponins predominant in raw material, less-glycosylated derivatives enriched after thermal processing, whole processed preparations, and metabolites generated after oral administration by the gut microbiota. The proposed processing-specific relevance rests on differences in constituent composition and pharmacokinetic context rather than on an assumed uniform anti-inflammatory advantage of processed preparations. The review evaluates how these evidence streams may relate to hepcidin-centered iron restriction by integrating thermal transformation, pharmacokinetic disposition, upstream inflammatory signaling, and gut–liver processes, while distinguishing established findings from indirect support and mechanistic hypotheses.

This narrative review was informed by literature searches conducted in PubMed, covering publications available up to August 2026. Search terms included combinations of “*Panax notoginseng*”, “Sanqi”, “processing”, “steaming”, “thermal processing”, “saponin”, “ginsenoside”, “hepcidin”, “*HAMP*”, “ferroportin”, “iron homeostasis”, “inflammation”, “IL-6”, “JAK/STAT”, “NF-κB”, and “gut microbiota”. Peer-reviewed primary studies and reviews were selected when they addressed processing-associated chemical transformation, saponin disposition, inflammatory or gut–liver pathways relevant to *HAMP* induction, or established mechanisms of hepcidin-mediated iron regulation. Studies of raw material, isolated compounds, processed preparations, and microbiota-derived metabolites were evaluated as distinct evidence streams and were not regarded as interchangeable evidence of a processing-dependent effect.

## 2. Raw-Material Variability and Traditional Thermal Processing

The phytochemical profile of *P. notoginseng* is influenced by both the composition of the starting material and its processing state. Geographic origin and cultivation conditions can affect the relative abundance of protopanaxadiol (PPD)- and protopanaxatriol (PPT)-type saponins, thereby influencing the precursor composition of raw materials [29,30,31,32]. Because processing-derived minor saponins arise from these precursors, baseline chemical variability may influence the composition of processed preparations.

Raw and thermally processed forms of *P. notoginseng* are traditionally regarded as having distinct applications, a principle often summarized as “sheng shu yi zhi” (differential efficacy based on processing) [5].

RPN is prepared by sun-drying or low-temperature air-drying harvested roots without deliberate heat treatment. As this method largely preserves the native profile of polar saponins, RPN is traditionally used for hemostatic purposes [2]. PPN is produced through various thermal treatments, predominantly steaming. Steamed *P. notoginseng* is traditionally used in blood-tonifying applications and during convalescence. Experimental steaming protocols vary in temperature and duration; for example, studies have evaluated conditions ranging from 100 to 130 °C for 5 h and from 105 to 120 °C for 2–10 h. During steaming, native polar saponins progressively shed sugar moieties and dehydrate, yielding rare lipophilic ginsenosides such as 20(S)-Rg3, 20(R)-Rg3, Rk1, and Rg5 [8,10]. These compositional changes may contribute to pharmacological differences between raw and steamed preparations, with the resulting effects depending on constituent composition, dose, and experimental context.

A related example is *P. notoginseng*-steamed chicken (PNSC), a medicinal food traditionally consumed by the Miao ethnic group in southeastern Yunnan and recognized locally for its tonic properties. In mouse models of postpartum blood-deficiency, acute hemorrhagic, and myelosuppressive anemia, freeze-dried PNSC soup improved hematological parameters and organ indices, with changes in JAK2/STAT5 signaling and hematopoiesis-related factors [6]. These findings provide preclinical support for the traditional blood-tonifying rationale of PNSC but do not establish erythropoietic recovery in patients.

Alternative thermal methods include stir-frying (130–200 °C) and honey-frying. Comparative studies demonstrate that varying thermal conditions produce distinct microstructural changes and differential patterns of saponin degradation [33]. Thus, processing-dependent changes in saponin composition may help explain differences between raw and processed preparations; however, the contributions of individual transformed saponins to oral exposure and therapeutic outcomes vary by compound and experimental context.

## 3. Chemical Transformation of Saponins During Processing

### 3.1. Saponin Profile of Raw P. notoginseng

The roots of *P. notoginseng* contain dammarane-type saponins of the PPD and PPT classes [34,35]. In raw roots, the saponin profile features PPT-type saponins, including ginsenoside Rg1 and notoginsenoside R1, alongside PPD-type saponins, with Rb1 as the most abundant individual compound [36]. These three compounds serve as standard pharmacological markers for quality evaluation [37], with notoginsenoside R1 distinguishing *P. notoginseng* from other *Panax* species [38]. PPD-type saponins (e.g., Rb1, Rd) carry sugar moieties at C-3 and C-20, whereas PPT-type saponins (e.g., Rg1, Re, R1) are glycosylated at C-6 and C-20. Notoginsenoside R1 carries a distinctive β-D-xylopyranosyl-(1→2)-β-D-glucopyranosyl chain at C-6, conferred by a specific xylosyltransferase during biosynthesis [39,40].

### 3.2. Thermal Transformation Pathways During Processing

**Figure 1:**
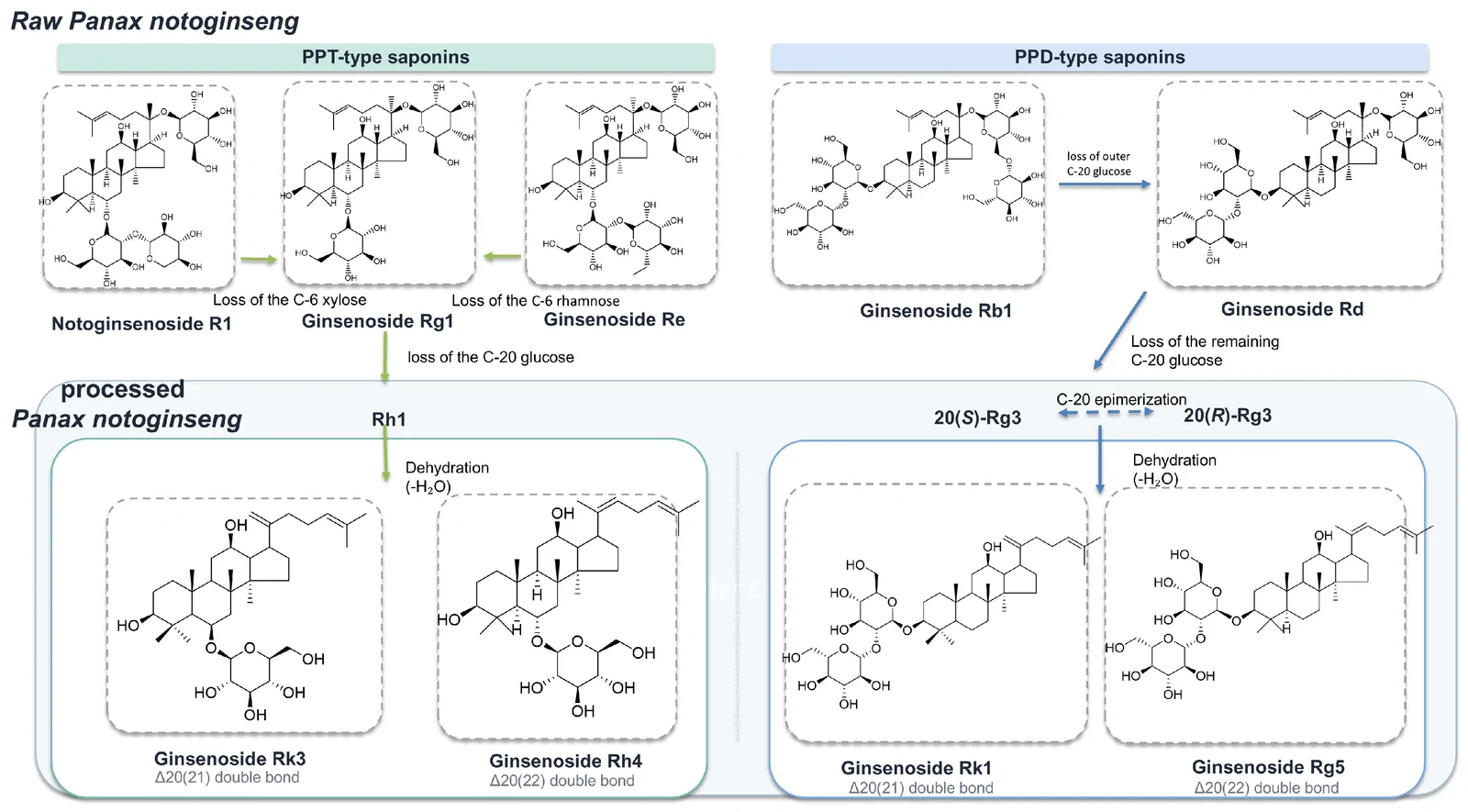
Representative structural relationships and thermal-processing transformations of major *Panax notoginseng* saponins (PNS). Major PPT-type saponins, including notoginsenoside R1 and ginsenosides Rg1 and Re, and PPD-type saponins, including ginsenosides Rb1 and Rd, may undergo deglycosylation, C-20 epimerization, and dehydration during thermal processing. Representative pathways include notoginsenoside R1 → Rg1 ← Re, Rg1 → Rh1 → Rk3/Rh4, and Rb1 → Rd → 20(S)-Rg3 ⇄ 20(R)-Rg3 → Rk1/Rg5. The scheme is representative rather than quantitative or exhaustive.

Thermal processing, particularly steaming, transforms native saponins through deglycosylation, dehydration, C-20 epimerization, demalonylation, and decarboxylation. Native polar saponins, including Rg1, Rb1, Rd, and notoginsenoside R1, together with thermally labile malonylated precursors, are progressively converted into less polar minor derivatives [41]. Malonyl-ginsenosides first undergo decarboxylation to acetylated intermediates and then deacetylation to the corresponding neutral ginsenosides [42]. Stepwise loss of glycosyl residues at C-3 and/or C-20 produces intermediates including 20(S)-Rg3 and 20(R)-Rg3; subsequent dehydration at C-20 yields dehydrated ginsenosides such as Rk1 and Rg5 [9,43]. In the studies cited, heating conditions altered the extent and product distribution of these transformations. Steaming at 140 °C for 2 h increased the yield of selected rare ginsenosides [7], whereas aspartic-acid impregnation before heating at 120 °C for 1 h increased the concentration of minor saponins [44]. Although thermal and microbial transformations can both reduce saponin glycosylation, thermal processing is a nonenzymatic, externally applied process with a distinct reaction environment and product profile. Representative structural relationships among major native saponins and processing-enriched minor ginsenosides are summarized in Figure 1. A comparison of the traditional applications, processing-associated compositional changes, and representative preclinical evidence for raw and thermally processed *P. notoginseng* is provided in Table 1. Deglycosylation and dehydration generally reduce saponin polarity and may influence membrane permeability and bioactivity in a congener- and model-dependent manner [43]. In the comparative study cited above, steaming produced higher concentrations of selected minor saponins than stir-frying, whereas stir-frying generated a broader metabolite spectrum [33].

## 4. Regulation of Iron Metabolism by Hepcidin

### 4.1. The Hepcidin–Ferroportin Axis: Modulating Iron Absorption and Recycling

Hepcidin, the peptide hormone that calibrates systemic iron homeostasis, acts by directly binding ferroportin (FPN), the sole known cellular iron exporter in mammals [18,20].

The hepcidin–FPN axis operates via a negative feedback loop. During iron excess or active inflammation, upregulated hepcidin binds FPN, triggering its internalization and lysosomal degradation, thereby sequestering iron intracellularly [17]. When erythropoietic demand rises, such as during hypoxia, or when iron availability is low, hepcidin transcription is downregulated, stabilizing plasma membrane FPN and sustaining iron efflux into the circulation [21].

This regulates both intestinal iron absorption and macrophage-mediated iron recycling. On the basolateral membrane of duodenal enterocytes, FPN mediates dietary iron transfer into the portal circulation. During chronic inflammation, hepcidin-mediated degradation of enterocytic FPN obstructs gastrointestinal iron absorption [52]. Within the reticuloendothelial system, FPN likewise enables macrophages to export iron reclaimed from senescent erythrocytes. Persistently elevated hepcidin depletes macrophage FPN, trapping recycled iron. This redistribution, characterized by hypoferremia with concurrent hyperferritinemia, illustrates how sustained hepcidin elevation reprograms systemic iron distribution [53].

### 4.2. Key Upstream Signals Regulating Hepcidin Expression

When systemic iron levels are elevated, liver endothelial cells produce bone morphogenetic protein (BMP) ligands that activate hepatocyte receptor complexes, triggering SMAD phosphorylation and nuclear translocation to upregulate hepcidin transcription [54]. Transmembrane serine protease 6 (TMPRSS6, also known as matriptase-2) attenuates this pathway by cleaving membrane-bound hemojuvelin (HJV), thereby downregulating BMP/SMAD signaling when iron is scarce. Pathogenic variants in the *TMPRSS6* gene disrupt this attenuation, resulting in unchecked hepcidin elevation. This finding highlights the centrality of BMP/SMAD signaling in hepcidin regulation [55].

Beyond iron-sensing pathways, inflammatory stress upregulates hepcidin synthesis. Interleukin-6 (IL-6) activates the JAK2/STAT3 cascade in hepatocytes; phosphorylated STAT3 then binds the hepcidin promoter, driving transcriptional upregulation that subsequently accelerates FPN degradation [22]. Chronic inflammation can engage NF-κB-dependent cytokine pathways that reinforce the inflammatory milieu associated with iron restriction [56]. In BV2 microglia, IL-6 neutralization altered STAT3 and SMAD1/5/9 phosphorylation and modified BMP6, TMPRSS6, and TfR2 expression, illustrating possible inflammatory–iron signaling crosstalk; its relevance to hepatocyte iron sensing requires direct confirmation [57].

### 4.3. Erythropoietic Signals and Negative Feedback Regulation: The Role of Erythroferrone

While iron-sensing and inflammatory pathways upregulate hepcidin to restrict iron availability, erythroferrone (ERFE) provides a counter-regulatory mechanism that helps coordinate iron delivery for erythropoiesis. ERFE, secreted by erythroblasts in response to erythropoietin (EPO), suppresses hepatic hepcidin transcription, thereby coordinating iron mobilization with hemoglobin synthesis requirements [19]. In settings of marked erythropoietic drive, such as ineffective erythropoiesis, elevated ERFE may suppress hepcidin despite systemic iron loading, prioritizing iron availability for erythropoiesis [58,59]. However, the contribution of ERFE to AI is context-dependent and should not be generalized from iron-loading anemia models. In anemia of critical illness, a form of inflammation-associated anemia, hepcidin remains elevated whereas EPO and ERFE levels are reduced, contributing to iron-restricted erythropoiesis [60].

## 5. Evidence from Processed Preparations and Processing-Enriched Saponins: Potential Relevance to Hepcidin-Related Pathways

Evidence from thermally processed *P. notoginseng* preparations is distinguished from evidence obtained using isolated saponins that can be enriched during processing. Evidence from raw material and microbiota-derived metabolites is also considered separately. Studies of individual compounds may inform constituent-specific mechanisms, but they do not by themselves demonstrate the pharmacological effects of a processed preparation or a processing-dependent effect on hepatic *HAMP*/hepcidin.

### 5.1. Inflammatory-Pathway Evidence Relevant to Hepcidin Regulation and Iron-Sensing Knowledge Gaps

Evidence relevant to the proposed mechanism derives from raw *P. notoginseng*, isolated saponins, *Panax notoginseng* saponins (PNS)-containing preparations, and thermally processed preparations; these sources should not be treated as interchangeable evidence for a processing-dependent effect. The available direct raw-versus-steamed comparison is limited to an LPS-stimulated RAW264.7 macrophage model, in which raw *P. notoginseng* suppressed TNF-α and IL-6 production more strongly than steamed material [47]. Thus, current evidence does not demonstrate that processing-enriched ginsenosides have uniformly greater anti-inflammatory activity than native saponins, nor does it establish a processing-dependent effect on hepatic *HAMP*/hepcidin. Rather, thermal processing changes the constituent composition and pharmacokinetic context of the preparation, which may alter the biological processes engaged under particular experimental conditions. PNS-containing preparations and isolated saponins have been reported to modulate inflammatory mediators or signaling pathways in diverse non-iron models, including JAK2/STAT3- and NF-κB-associated pathways [61,62,63,64,65,66,67,68,69,70]. These findings support the possibility that inflammatory inputs relevant to *HAMP* induction may be altered. However, pathway inhibition in macrophages, neural cells, podocytes, or other non-hepatocyte systems does not demonstrate altered hepatic *HAMP* transcription, circulating hepcidin, FPN abundance, or systemic iron availability.

Studies of thermally processed *P. notoginseng* provide preclinical support for the traditional blood-tonifying rationale, but their hematological findings require context-specific interpretation. In blood-deficiency, renal-anemia, and cyclophosphamide-anemia models, processed preparations were associated with improved hematological indices and selected hematopoietic or tissue-injury endpoints [48,49,50]. In one blood-deficiency study, Rk3 and 20(S)-Rg3 were associated with increased blood parameters, hepatic ferrochelatase, and heme [51]. These findings may reflect effects on hematopoietic support, heme biosynthesis, or protection from anemia-associated tissue injury. Because these studies did not directly assess hepatic *HAMP*/hepcidin, circulating hepcidin, FPN abundance, or iron flux, the reported hematological improvements should not be interpreted as correction of hepcidin-mediated iron restriction. The study reporting TNF and Toll-like receptor nodes as candidate targets should likewise be interpreted cautiously because target-expression validation was performed in an Alzheimer’s disease brain model rather than an anemia model [71].

These hematological observations do not identify a direct route to *HAMP* transcription. Bone-related reports suggesting that PNS influence BMP-related or TGF-β1/SMAD3 signaling should not be interpreted as evidence of direct modulation of the hepatic BMP6/SMAD iron-sensing axis [72,73]. In contrast, hepatocyte-based studies establish IL-6/STAT3-dependent *HAMP* induction [22,74]. At the *HAMP* promoter, the BMP-responsive element BMP-RE1 links HFE2/BMP-dependent basal *HAMP* expression to its responsiveness to IL-6 [75], whereas a modeling analysis suggests that SMAD and STAT act at adjacent promoter elements and may compete for RNA polymerase II recruitment [76]. Inflammatory stimuli can also engage activin B–SMAD1/5/8 signaling in hepatocytes [77]. Thus, PNS- or ginsenoside-associated anti-inflammatory activity may be relevant to upstream inflammatory inputs, whereas the available bone-related and non-hepatocyte studies do not address direct modulation of hepatic iron-sensing modules. The proposed mechanism is most relevant to inflammation-associated iron restriction and should not be generalized to disorders dominated by BMP6-dependent iron sensing or TMPRSS6-related defects [54,55,78] or to settings in which erythropoietic signals substantially influence hepcidin regulation [19,58,59,60].

### 5.2. Processing-Associated Pharmacokinetic Considerations and Hepatic Exposure

The potential relevance of processed PNS to hepcidin-related pathways may depend on both constituent composition and pharmacokinetic disposition. Native, highly glycosylated saponins, such as Rb1 and Rg1, generally show limited intestinal permeability and oral bioavailability [79,80]. Thermal processing enriches less-glycosylated derivatives, including Rg3, Rk1, and Rg5, which can differ from their native precursors in membrane permeability and systemic exposure [11]. Differences in systemic exposure between raw and processed preparations reflect not only absorption and metabolism, but also differences in constituent profiles and administered amounts. Reduced polarity or altered systemic exposure alone should therefore not be interpreted as evidence of improved oral bioavailability or pharmacologically relevant hepatic exposure.

Following intestinal absorption, saponin metabolites enter the portal circulation and may be delivered to the liver, the principal site of hepcidin synthesis. Reviews of Rg3 pharmacokinetics describe rapid absorption and low circulating concentrations, a pattern compatible with tissue distribution [81]. Radioisotope biodistribution work has also reported hepatic distribution of orally administered ginsenosides [82]. These observations make hepatic exposure plausible, but they do not establish the hepatic concentrations achieved after administration of processed preparations or show that such concentrations are sufficient to influence hepatocyte *HAMP* expression or iron sensing.

Steamed *P. notoginseng* saponins have been evaluated in a murine model of cyclophosphamide-induced anemia [49]. Improvements in hemoglobin-related outcomes and indices of gut–liver injury, together with changes in hepatic signaling, are consistent with altered pharmacological activity after processing. They may also reflect hematopoietic support, tissue protection, or recovery from treatment-related injury. Because hepcidin, FPN abundance, and iron flux were not measured, this study cannot distinguish among these explanations or demonstrate correction of hepcidin-mediated iron restriction.

### 5.3. Gut–Liver Axis and Potential Implications for Iron Availability

Elevated hepcidin binds FPN on the basolateral membrane of duodenal enterocytes and on macrophages, inducing its internalization and degradation; this restricts dietary iron export and macrophage iron recycling, thereby contributing to functional iron deficiency [52]. In the same cyclophosphamide-induced anemia model, steamed *P. notoginseng* saponins were associated with improved histopathological findings in the liver and small intestine [49]. These observations provide a gut–liver injury context for the proposed framework; however, because hepatic *HAMP* expression, circulating hepcidin, FPN abundance, and iron flux were not assessed, they do not demonstrate correction of hepcidin-mediated iron restriction. Experimental liver injury can perturb hepcidin and iron metabolism [83], whereas reduced DMT1 expression in intestinal epithelial cells during inflammatory bowel disease illustrates how intestinal injury and inflammation can compromise dietary iron uptake [84]. If processed saponins are shown in vivo to lower hepcidin, preservation of FPN on enterocytes and macrophages could facilitate intestinal iron export and macrophage iron recycling and may improve iron availability for erythropoiesis.

Table 2 maps experimental observations on anti-inflammatory or microbiota-related actions of isolated ginsenosides that can be enriched during thermal processing. As stated in the Table 2 Note, these studies used non-iron models and identify candidate inflammatory or gut–liver contexts for the hypothesis; they are not direct evidence of hepcidin-mediated iron regulation by processed *P. notoginseng* preparations. The proposed framework is therefore most relevant to inflammatory settings in which gut-derived or systemic inflammatory signals contribute to *HAMP* induction. It does not predict that processed preparations will alter hepcidin under all physiological or pathological conditions, nor that such preparations will override BMP6-dependent iron sensing, TMPRSS6-related regulation, or erythropoietic control of hepcidin. Support for a processing-specific effect would require matched raw-versus-processed interventions with defined processing conditions, quantified constituent profiles, comparable administered doses, pharmacokinetic characterization, and concordant changes in hepatic *HAMP*/hepcidin, FPN abundance, iron flux, and iron-related outcomes. Conversely, changes in the saponin profile or inflammatory markers without corresponding changes in these iron-regulatory endpoints would not support a hepcidin-mediated mechanism.

## 6. The Gut–Liver Axis and Microbiota-Driven Saponin Metabolism: Potential Relevance to Hepcidin-Related Pathways

The microbiota-related processes discussed in this section occur after oral administration and should be distinguished from external thermal processing. They may modify the exposure and biological context of ingested saponins, but do not constitute direct evidence for thermally processed *P. notoginseng* preparations.

### 6.1. Gut Microbiota-Mediated Biotransformation

Within the intestinal lumen, primary ginsenosides, such as Rb1 and Rg1, can be exposed to microbial enzymes and undergo structural biotransformation that may influence host pharmacokinetics [87]. Bacterial β-glucosidases can hydrolyze glycosidic bonds in selected saponin substrates; for example, EfD08 from *Enterococcus faecalis* hydrolyzed multiple steroidal saponins and showed activity toward Rb1 and Rc in vitro [88].

Microbiota-dependent transformation may partially converge with external thermal processing in terms of deglycosylation outcomes. However, it is not a biochemical equivalent of thermal processing because the catalysts, reaction conditions, transformation pathways, product profiles, and host dependence differ. Controlled microbial or enzymatic systems can selectively modify the glycosylation patterns of saponin substrates [89,90,91], and controlled fermentation can alter the chemical composition of *Panax* preparations [92]. However, neither engineered process should be extrapolated to host intestinal microbial biotransformation.

Microbial deglycosylation can generate more lipophilic metabolites, which may facilitate intestinal absorption and hepatic exposure [12,13]. Current biotransformation and pharmacokinetic studies do not resolve whether microbiota-derived metabolites directly influence hepatocyte signaling or *HAMP* transcription. The extent of microbial biotransformation may vary among individuals according to host intestinal microbiota composition. Ex vivo mixed fecal-microbiota cultures have identified substantial interindividual variation in Rb1 biotransformation [93]. This variability may contribute to differences in systemic exposure to secondary saponin metabolites. Microbially induced structural modifications may also alter saponin–receptor interactions, with potential consequences for downstream metabolic responses [94].

### 6.2. Microbiota–Immune Crosstalk and Potential Implications for Hepcidin Regulation

PNS may indirectly reduce inflammatory inputs implicated in hepcidin induction through potential effects on intestinal barrier function and LPS-driven signaling. The intestinal epithelium functions as both a physical barrier and an immunological sensor, and its dysfunction can contribute to systemic endotoxemia and chronic inflammation. In experimental colonic models, ginsenoside Rb1 increased the expression of the tight-junction proteins ZO-1 and occludin and the adherens-junction protein E-cadherin, potentially through modulation of VDR, PPARγ, and NF-κB signaling pathways [26]. These barrier-associated effects may limit bacterial translocation and endotoxin leakage into the circulation.

Improved barrier function may limit LPS translocation into the portal circulation. In duckling models, ginsenoside Rg1 was associated with a reduced abundance of LPS-producing Gram-negative bacteria, potentially decreasing the luminal endotoxin pool available for translocation [95]. Collectively, microbiota- and barrier-related actions of PNS constituents may limit gut-to-liver exposure to microbial pathogen-associated molecular patterns and attenuate LPS-induced TLR4 signaling [25].

A reduction in circulating LPS could attenuate hepatic Kupffer-cell activation and downstream TLR4/NF-κB signaling, thereby reducing pro-inflammatory cytokine production. CK has been reported to alleviate mucosal inflammation while modulating gut microbiota and reducing pro-inflammatory cytokines, including TNF-α, IL-6, IL-1β, and IL-17 [96]. Because IL-6 is a major driver of inflammation-induced hepcidin transcription through hepatocyte JAK2/STAT3 signaling, reduced macrophage-derived IL-6 could attenuate an important upstream input implicated in hepcidin induction [22]. By restoring barrier integrity, limiting LPS translocation, and suppressing macrophage-derived IL-6, PNS may indirectly reduce inflammatory inputs implicated in hepcidin induction. Whether these barrier- and inflammation-related observations translate into lower *HAMP*/hepcidin expression or improved iron availability in vivo has not been tested in interventions with processed *P. notoginseng* preparations.

**Figure 2:**
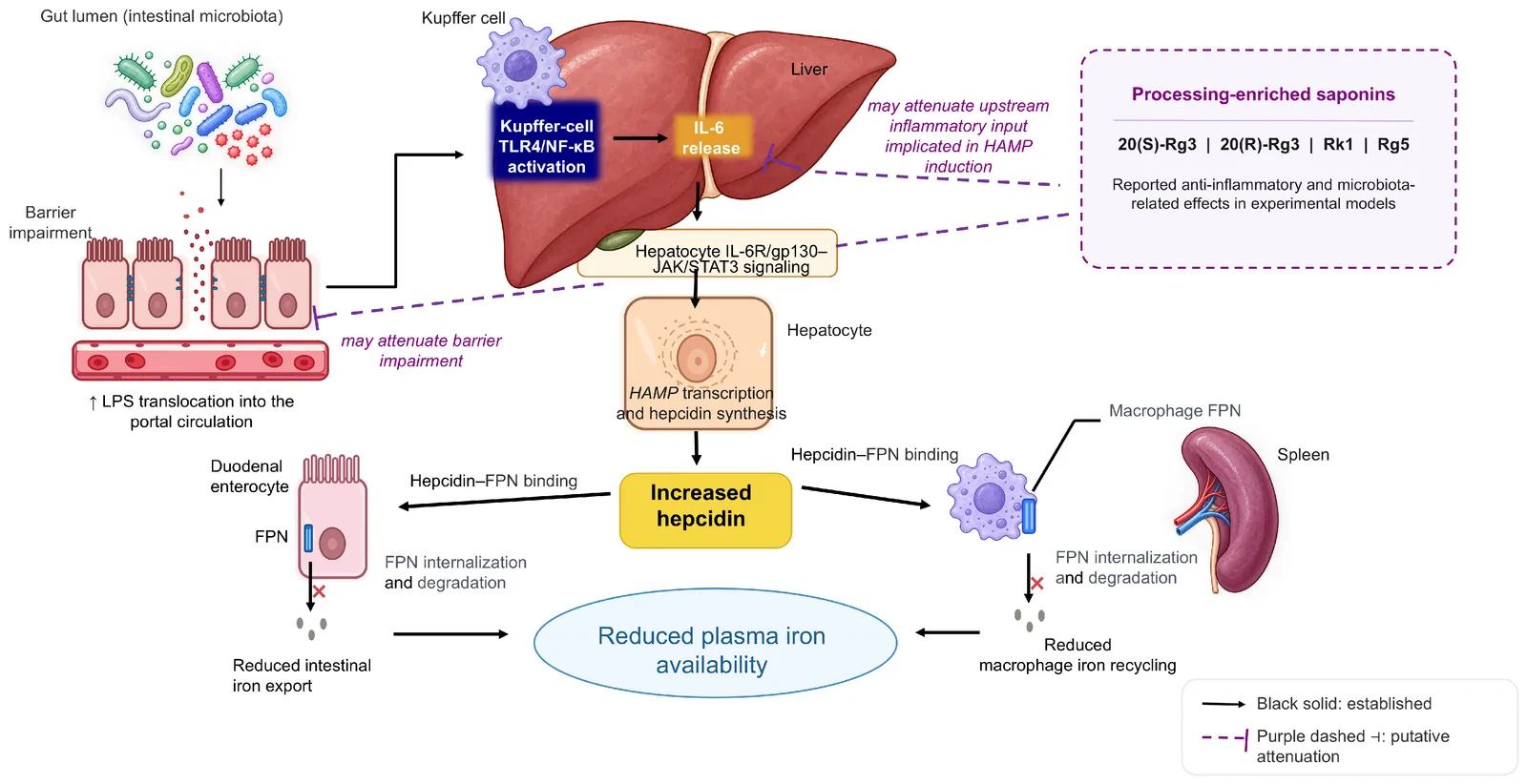
Hepcidin-centered regulation of plasma iron availability via the gut–liver axis and putative modulatory sites of processing-enriched PNS. Under conditions of intestinal barrier impairment, luminal microbiota-derived lipopolysaccharide (LPS) can translocate into the portal circulation and activate TLR4/NF-κB signaling in hepatic Kupffer cells, thereby promoting IL-6 production. IL-6 acts on hepatocytes through IL-6R/gp130–JAK/STAT3 signaling to induce *HAMP* transcription and hepcidin synthesis. Circulating hepcidin subsequently binds to ferroportin (FPN) on duodenal enterocytes and splenic macrophages, triggering FPN internalization and degradation, which reduces intestinal iron export and macrophage-mediated iron recycling and ultimately decreases plasma iron availability. Solid black arrows denote established relationships. Red crosses indicate the inhibitory consequences of hepcidin-mediated FPN internalization and degradation, namely reduced intestinal iron export and macrophage iron recycling. Purple dashed lines with T-shaped termini denote putative attenuation of TLR4/NF-κB–IL-6 inflammatory input or support of intestinal barrier function by processing-enriched rare saponins, including 20(S)-Rg3, 20(R)-Rg3, Rk1, and Rg5. These saponin-related links are mechanistic inferences; direct in vivo evidence that processed *P. notoginseng* saponins reduce hepatic *HAMP*/hepcidin expression, preserve FPN abundance, or improve iron availability remains to be established.

The established gut–liver inflammatory route and the putative upstream sites at which processing-enriched saponins may act are summarized in Figure 2.

## 7. Conclusions

Thermal processing of *P. notoginseng* alters the native saponin profile through deglycosylation, dehydration, and C-20 epimerization, enriching less-glycosylated minor ginsenosides, including 20(S)-Rg3, 20(R)-Rg3, Rk1, and Rg5. This compositional shift provides a plausible chemical basis for differences between raw and processed preparations, but it does not establish a uniform pharmacological advantage of the processed form.

The proposed framework rests on two established biological relationships: inflammation-associated IL-6–JAK2/STAT3 signaling can induce hepatic *HAMP* expression, and hepcidin restricts iron export by promoting ferroportin internalization and degradation. Whether processing-associated changes in *P. notoginseng* composition modify these pathways in vivo remains uncertain. Evidence from processed preparations, isolated processing-enriched ginsenosides, and microbiota-derived metabolites provides indirect support for possible effects on upstream inflammatory or gut–liver processes; however, these evidence streams cannot be treated as interchangeable evidence of reduced hepcidin expression or improved iron availability.

A decisive test of the processing-specific hypothesis would require matched raw-versus-processed interventions with defined processing conditions, quantified constituent profiles, comparable administered doses, and pharmacokinetic characterization. Such studies should directly assess hepatic *HAMP* expression, circulating hepcidin, ferroportin abundance, iron flux, and iron-related outcomes alongside inflammatory and erythropoietic indices. Conversely, changes in saponin composition, cytokine levels, or hematological indices without concordant changes in these iron-regulatory endpoints would not support a hepcidin-mediated mechanism. This review therefore defines a testable framework linking processing-associated chemical changes to potential hepcidin-related pathways, rather than inferring a direct iron-regulatory effect from anti-inflammatory activity alone.

## Figures and Tables

**Table 1:** Evidence map of raw and thermally processed *P. notoginseng*: preparation, chemical features, reported outcomes, and limits of inference.

Form and Preparation	Traditional Application	Processing-Associated and Compositional Features	Representative Experimental Evidence	Key Limitation
RPN	Traditionally used for hemostasis, resolution of blood stasis, and relief of trauma-related swelling and pain [1,2].	Sun-dried or low-temperature air-dried without deliberate thermal processing. Major saponins include notoginsenoside R1 and ginsenosides Rg1, Rb1, Rd, and Re; Rg3, Rk1, and Rg5 are absent or present at low abundance [42,45,46].	In LPS-stimulated RAW264.7 macrophages, RPN suppressed TNF-α and IL-6 production more strongly than steamed material. Notoginsenoside R1 and ginsenosides Rg1, Re, and Rb1 were associated with the observed anti-inflammatory activity [47].	Cell-based comparison only; it did not assess systemic exposure, hepatic *HAMP*/hepcidin, FPN, or iron-related outcomes.
PPN	Traditionally used for blood tonification and convalescent support [5,48]	Thermal conditions vary among studies. Representative examples include steaming at 140 °C for 2 h and aspartic-acid impregnation followed by heating at 120 °C for 1 h [7,44]. Deglycosylation, dehydration, and C-20 epimerization reduce major native saponins and enrich minor ginsenosides, including 20(S)-Rg3, 20(R)-Rg3, Rk1, and Rg5 [7,8,9,10,42,45,46]	In blood-deficiency, renal-anemia, and cyclophosphamide-anemia models, processed preparations were associated with improved hematological indices and selected hematopoietic or tissue-injury endpoints [48,49,50]. Rk3 and 20(S)-Rg3 were associated with increased blood parameters, hepatic ferrochelatase, and heme in one study [51].	Processing conditions, constituent profiles, doses, and disease models differ. *HAMP*/hepcidin, FPN abundance, and iron flux were not directly assessed.

Note: This table is an evidence-map comparison, not a head-to-head efficacy comparison. Traditional applications are provided for context and do not establish clinical efficacy. The raw-versus-steamed anti-inflammatory comparison is limited to a cell model, whereas findings for processed preparations derive from heterogeneous animal models. Therefore, between-row differences should not be interpreted as superiority of either form or as evidence of altered hepatic *HAMP* expression, circulating hepcidin, ferroportin abundance, or iron availability.

**Table 2:** Indirect evidence from processing-enriched ginsenosides relevant to hepcidin-associated pathways.

Ginsenoside	Model and Reported Exposure	Reported Upstream Effect	Indirect Relevance to the Hepcidin-Related Framework
20(*S*)-Rg3 [65]	RAW264.7: 20(S)-Rg3, 0–50 μM, 1 h pretreatment; LPS, 1 μg/mL, 24 h. HaCaT: 20(S)-Rg3, 0–25 μM, 24 h; TNF-α, 20 ng/mL, added after 1 h when indicated.	Reduced NO, ROS, PGE_2_, COX-2, MMP-9, and several inflammatory mediators	Inflammatory-mediator context
20(*S*)-Rg3 [66]	LPS-activated RAW264.7 macrophages; Rg3, 50 μM for 1 h before LPS, 100 ng/mL for 12 or 24 h	Reduced NO, TNF-α, and IL-6; altered eicosanoid profiles	IL-6-associated inflammatory input
20(*R*)-Rg3 [67]	MCAO/R rats and HT22 OGD/R cells; rats received Rg3, 5, 10, or 20 mg/kg, intraperitoneally at −12, 0, and +12 h	Reduced TLR4, MyD88, p-NF-κB p65, IL-6, IL-1β, IFN-γ, and TNF-α	TLR4/NF-κB-associated inflammatory input
20(*R*)-Rg3 [68]	Male ICR mice with APAP-induced injury: APAP, 250 mg/kg, single dose; 20(R)-Rg3 pretreatment, 7 days.	Attenuated oxidative stress, inflammatory response, necrosis, and apoptosis through PI3K/AKT-associated signaling	Hepatic inflammatory context
Rk1 [69]	RAW264.7 cells: Rk1 at 10, 20, or 40 μM; LPS at 1 μg/mL.	Reduced JAK2/STAT3 phosphorylation and pro-inflammatory responses	JAK2/STAT3-associated inflammatory input
Rk1 [24]	LPS-induced acute kidney injury in C57BL/6 mice and MPC-5 cells; mice received Rk1, 10 or 20 mg/kg orally, after LPS, 10 mg/kg intraperitoneally	Reduced renal injury, inflammation, and podocyte apoptosis; inactivated JAK2/STAT3 and NF-κB signaling	JAK2/STAT3- and NF-κB-associated inflammatory input
Rg5 [70]	LPS-stimulated alveolar macrophages and murine lung-inflammation model; Rg5, 10 mg/kg in vivo	Reduced LPS–TLR4-related signaling, IRAK/IKK–NF-κB activation, and inflammatory indices	LPS–TLR4/NF-κB-associated inflammatory input
Rg5 [85]	HFD-induced NAFLD in C57BL/6J mice; Rg5, 50 or 100 mg/kg/day by gavage for 12 weeks	Improved liver injury and metabolic phenotypes; altered gut microbiota and LKB1/AMPK/mTOR signaling	Gut–liver and inflammatory context
Rg5 [86]	db/db mice; Rg5, approximately 90 mg/kg/day by gavage for 4 weeks	Improved glycemic and metabolic outcomes and modified gut microbiota dysbiosis	Microbiota- and metabolic-context evidence

Note: This table summarizes studies of isolated ginsenosides that can be enriched during thermal processing, rather than chemically characterized processed *P. notoginseng* preparations. All listed studies used non-iron experimental models and did not directly assess hepatic *HAMP* expression, circulating hepcidin, ferroportin abundance, iron flux, or systemic iron availability. The reported findings therefore provide indirect support for possible upstream inflammatory or gut–liver processes, but do not establish a processing-dependent effect on hepcidin-mediated iron regulation.

## Data Availability

No new data were created or analyzed in this study. Data sharing is not applicable to this article.

## Abbreviations

The following abbreviations are used in this manuscript:

AI	Anemia of inflammation
BMP	Bone morphogenetic protein
CK	Compound K
EPO	Erythropoietin
ERFE	Erythroferrone
FPN	Ferroportin
*HAMP*	Hepcidin antimicrobial peptide
HJV	Hemojuvelin
IL-6	Interleukin-6
JAK2	Janus kinase 2
LPS	Lipopolysaccharide
NF-κB	Nuclear factor kappa B
PNS	*Panax notoginseng* saponins
PPD	Protopanaxadiol
PPN	Processed *Panax notoginseng*
PPT	Protopanaxatriol
RPN	Raw *Panax notoginseng*
SMAD	Small mothers against decapentaplegic
STAT3	Signal transducer and activator of transcription 3
TCM	Traditional Chinese medicine
TLR4	Toll-like receptor 4
*TMPRSS6*	Transmembrane serine protease 6
TNF-α	Tumor necrosis factor alpha

## References

[1] Zhang X., Li C., Wang G., Francis O.B., Wang H., Sun A., Wu H., Yang X., Dong P., Zheng W. (2025). The integration of spear and shield: A panoramic analysis of the blood circulation-promoting and hemostatic effects of Panax notoginseng. Chin. Med..

[2] Liu J., Zhang G., Wang Y., Hao Z., Xue J., Lu Y., Fan W., Wang C., Shi J. (2024). Screening and verification of hemostatic effective components group of Panax Notoginseng based on spectrum-effect relationships. J. Ethnopharmacol..

[3] Wei G., Zhang G., Li M., Zheng Y., Zheng W., Wang B., Zhang Z., Zhang X., Huang Z., Wei T. (2024). Panax notoginseng: Panoramagram of phytochemical and pharmacological properties, biosynthesis, and regulation and production of ginsenosides. Hortic. Res..

[4] Gao M., Cao X., Wei S., Huang X., Ouyang H., Chang Y., Shi R., He J. (2022). Quantitative comparison and chemical profile of different botanical parts of Panax notoginseng from different regions. Front. Nutr..

[5] Zeng X., Zheng Y., Chen P., Nie Y., Mo J., Yan R., Zhou T., Cheng H., Jiang J. (2026). Processing-driven chemical transformations and pharmacological shifts in Panax notoginseng. J. Ethnopharmacol..

[6] Chen Z., Chen X., Guo L., Cui X., Qu Y., Yang X., Liu Y., Wang C., Yang Y. (2023). Effect of different cooking methods on saponin content and hematopoietic effects of Panax notoginseng-steamed chicken on mice. J. Ethnopharmacol..

[7] Fan W., Liao Q., Fan L., Li Q., Liu L., Wang Z., Mei Y., Li L., Yang L., Wang Z. (2024). An innovative processing driven efficient transformation of rare ginsenosides enhances anti-platelet aggregation potency of notoginseng by integrated analyses of processing-(chemical) profiling-pharmacodynamics. J. Ethnopharmacol..

[8] Ning Y., Wang N., Tian S., Liang Y., Ma J., Cui X. (2026). Steam processing of Panax notoginseng with single-marker quantification: A green industrial standardization strategy. BMC Chem..

[9] Zhao W.J., Han L.L., Li T., Lee J., Zhao Y.Q. (2024). Effects of steaming process on rare saponins and efficacy of Panax ginseng, Panax notoginseng and Panax quinquefolium. Chin. Herb. Med..

[10] Ma D., Wang J., Yin G., Wang L., Jin Y., Huang Y., Bi K., Lu Y., Wang T. (2022). The study of steaming durations and temperatures on chemical characterization, neuroprotective and antioxidant activities of Panax notoginseng. Evid. Based Complement. Altern. Med..

[11] Dong J.J., Yin Z.Z., Su L.L., Yu M.T., Wang M., Li L., Mao C.Q., Lu T.L. (2021). Comparative pharmacokinetic analysis of raw and steamed Panax notoginseng roots in rats by UPLC-MS/MS for simultaneously quantifying seven saponins. Pharm. Biol..

[12] Morshed M.N., Akter R., Karim M.R., Iqbal S., Kang S.C., Yang D.C. (2024). Bioconversion, pharmacokinetics, and therapeutic mechanisms of ginsenoside compound K and its analogues for treating metabolic disease. Curr. Issues Mol. Biol..

[13] Zheng Y., Su W., Liu Y., Zhang W., Zeng X. (2024). Gut microbiota-mediated metabolism of Panax notoginseng saponins and its role in pharmacokinetics and pharmacodynamics. Tradit. Med. Res..

[14] Shin N.R., Bose S., Choi Y., Kim Y.M., Chin Y.W., Song E.J., Nam Y.D., Kim H. (2021). Anti-obesity effect of fermented Panax notoginseng is mediated via modulation of appetite and gut microbial population. Front. Pharmacol..

[15] Yan S., Wei P.-C., Chen Q., Chen X., Wang S.-C., Li J.-R., Gao C. (2018). Functional and structural characterization of a β-glucosidase involved in saponin metabolism from intestinal bacteria. Biochem. Biophys. Res. Commun..

[16] Mo Y., He X., Cui H., Cheng Y., Zhou M., Cui X., Zhang T. (2025). Gut microbiota: A new key of understanding for Panax notoginseng against multiple disorders and biotransformation. J. Ethnopharmacol..

[17] Nemeth E., Ganz T. (2021). Hepcidin–ferroportin interaction controls systemic iron homeostasis. Int. J. Mol. Sci..

[18] Kesharwani P., Dash D., Koiri R.K. (2025). Deciphering the role of hepcidin in iron metabolism and anemia management. J. Trace Elem. Med. Biol..

[19] Ganz T. (2025). Systemic iron metabolism. Adv. Exp. Med. Biol..

[20] Silvestri L., Pettinato M., Furiosi V., Bavuso Volpe L., Nai A., Pagani A. (2023). Managing the dual nature of iron to preserve health. Int. J. Mol. Sci..

[21] Ginzburg Y.Z. (2023). Hepcidin and its multiple partners: Complex regulation of iron metabolism in health and disease. Vitam. Horm..

[22] Kowdley K.V., Gochanour E.M., Sundaram V., Shah R.A., Handa P. (2021). Hepcidin signaling in health and disease: Ironing out the details. Hepatol. Commun..

[23] Jang W.Y., Hwang J.Y., Cho J.Y. (2023). Ginsenosides from Panax ginseng as key modulators of NF-κB signaling are powerful anti-inflammatory and anticancer agents. Int. J. Mol. Sci..

[24] Ma X., Pang L., Shi F., Guan B. (2025). Ginsenoside Rk1 exerts protective effects of LPS-induced podocyte apoptosis and inflammation by inactivating JAK2/STAT3 and NF-κB pathways. Drug Chem. Toxicol..

[25] Xu Y., Wang N., Tan H.Y., Li S., Zhang C., Feng Y. (2021). Gut-liver axis modulation of Panax notoginseng saponins in nonalcoholic fatty liver disease. Hepatol. Int..

[26] Zhou Y., Xiong X., Cheng Z., Chen Z., Wu S., Yu Y., Liu Y., Chen G., Li L. (2024). Ginsenoside Rb1 alleviates DSS-induced ulcerative colitis by protecting the intestinal barrier through the signal network of VDR, PPARγ and NF-κB. Drug Des. Dev. Ther..

[27] Kumar M., Leon Coria A., Cornick S., Petri B., Mayengbam S., Jijon H.B., Moreau F., Shearer J., Chadee K. (2020). Increased intestinal permeability exacerbates sepsis through reduced hepatic SCD-1 activity and dysregulated iron recycling. Nat. Commun..

[28] Abdulgayoom M., Alshurafa A., Kaddoura R., Al-Mashdali A.F., Mohamed S.F., Yassin M.A. (2026). Anemia of chronic disease revisited: Hepcidin dysregulation, erythropoietic failure, and emerging roles of SGLT2 inhibition. Eur. J. Haematol..

[29] Zhan P., Wang F., Xia P., Zhao G., Wei M., Wei F., Han R. (2022). Assessment of suitable cultivation region for Panax notoginseng under different climatic conditions using MaxEnt model and high-performance liquid chromatography in China. Ind. Crops Prod..

[30] Liu Y., Wang Y., Tian G., Yang S., Chen J., Zi S., Fan W., Ma Q., Liu J., Zhang Z. (2025). Panax notoginseng planted under coniferous forest: Effects on soil health and the soil microbiome. Forests.

[31] Liang Z.W., Guan Y.H., Lv Z., Yang S.C., Zhao M., Chen J.W. (2026). Metagenomics reveals an interaction among rhizosphere microbial community, soil properties and active ingredients in a medicinal crop Panax notoginseng. J. Ginseng Res..

[32] Xu Y., Zhu M., Feng Y., Xu H. (2023). Panax notoginseng–microbiota interactions: From plant cultivation to medicinal application. Phytomedicine.

[33] Xu C., Fang Q., Cui H., Lin Y., Dai C., Li X., Tu P., Cui X. (2024). Comparison of the components of fresh Panax notoginseng processed by different methods and their anti-anemia effects on cyclophosphamide-treated mice. J. Ethnopharmacol..

[34] Xie W.B., Xia L., Li H., Li W., Cao Y., Huang Y., Lei F.H. (2022). Preparation of modified rosin bonded silica high performance liquid chromatographic stationary phase and separation of Panax notoginseng saponins. Chin. J. Chromatogr..

[35] Tian Y., Fan J., Wang R., Ding R., Zhao C., Xu J., Zhang Q., Nan T., Li X., Ye M. (2024). Triterpenoid saponins from the roots of Panax notoginseng with protective effects against APAP-induced liver injury. Fitoterapia.

[36] Wang J.R., Yau L.F., Gao W.N., Liu Y., Yick P.W., Liu L., Jiang Z.H. (2014). Quantitative comparison and metabolite profiling of saponins in different parts of the root of Panax notoginseng. J. Agric. Food Chem..

[37] He S., Zhang S.M., Xing Y.D., Zang T.Y., Xu X.F., Hua M.Z., Wang Z.X., Zhao L.J., Shi R., Wang S. (2026). Quantitative analysis of fifteen saponins in the roots and leaves of Panax notoginseng under different cultivation models using UPLC-Q-Orbitrap HRMS. J. Agric. Food Res..

[38] Tian L.X., Li J.H., Zhang L., Ahmad B., Huang L.F. (2021). Discrimination of five species of Panax genus and their geographical origin using electronic tongue combined with chemometrics. World J. Tradit. Chin. Med..

[39] Liu R., Wu X., Jiang Z., Liu X., Zhang Y., Zhao H., Gao J., Gao W., Hu Y., Huang L. (2024). Characterization of a xylosyltransferase from Panax notoginseng catalyzing ginsenoside 2′-O-glycosylation in the biosynthesis of notoginsenosides. J. Nat. Prod..

[40] Hou M., Nie F., Zhao J., Ju Z., Yang L., Wang Q., Zhao S., Wang Z. (2023). New glycosyltransferases in Panax notoginseng perfect main ginsenosides biosynthetic pathways. J. Agric. Food Chem..

[41] Wang K.H., Liu J.Y., Ma L.J., Zhang F.X., Wan J.B. (2026). The rise of dehydrated ginsenosides: Phytochemistry and pharmacology. J. Adv. Res..

[42] Fan W., Yang Y., Li L., Fan L., Wang Z., Yang L. (2022). Mass spectrometry-based profiling and imaging strategy, a fit-for-purpose tool for unveiling the transformations of ginsenosides in Panax notoginseng during processing. Phytomedicine.

[43] Jia W., Zhang H., Cheng J., Zhang X., Han C., He Y., Lu H., Liu H., Li C., Ma H. (2026). Extraction, preparation, pharmacological activities, and potential applications of ginsenosides Rk1 and Rg5. J. Ethnopharmacol..

[44] Cui Y., Wu J., Zhang D., Li D., Zhang J., Li W., Wang C., Yuan C., Liu Z. (2024). Changes in chemical components and hepatoprotective effect of red Panax notoginseng processed by aspartic acid impregnation treatment. J. Sci. Food Agric..

[45] Xiong Y., Chen L., Man J., Hu Y., Cui X. (2019). Chemical and bioactive comparison of Panax notoginseng root and rhizome in raw and steamed forms. J. Ginseng Res..

[46] Wang D., Liao P.-Y., Zhu H.-T., Chen K.-K., Xu M., Zhang Y.-J., Yang C.-R. (2012). The processing of Panax notoginseng and the transformation of its saponin components. Food Chem..

[47] Zhang Z., Chen L., Cui X., Zhang Y., Hu Y., Wang C., Xiong Y. (2019). Identification of anti-inflammatory components of raw and steamed Panax notoginseng root by analyses of spectrum-effect relationship. RSC Adv..

[48] Gao M., Zhang Z., Zhang Y., Li M., Che X., Cui X., Wang M., Xiong Y. (2022). Steamed Panax notoginseng attenuates renal anemia in an adenine-induced mouse model of chronic kidney disease. J. Ethnopharmacol..

[49] Xu C., Cui H., Fang Q., Tu P., Cui X. (2025). Steamed Panax notoginseng saponins ameliorate cyclophosphamide-induced anemia by attenuating gut–liver injury and activating the cAMP/PI3K/AKT signaling pathway. Nutrients.

[50] Zhang Z., Zhang Y., Gao M., Cui X., Yang Y., van Duijn B., Wang M., Hu Y., Wang C., Xiong Y. (2019). Steamed Panax notoginseng Attenuates Anemia in Mice With Blood Deficiency Syndrome via Regulating Hematopoietic Factors and JAK-STAT Pathway. Front. Pharmacol..

[51] Xiong Y., Hu Y., Chen L., Zhang Z., Zhang Y., Niu M., Cui X. (2018). Unveiling Active Constituents and Potential Targets Related to the Hematinic Effect of Steamed Panax notoginseng Using Network Pharmacology Coupled with Multivariate Data Analyses. Front. Pharmacol..

[52] Karaskova E., Pospisilova D., Velganova-Veghova M., Geryk M., Volejnikova J., Holub D., Hajduch M. (2021). Importance of hepcidin in the etiopathogenesis of anemia in inflammatory bowel disease. Dig. Dis. Sci..

[53] Chang J., Debreli Coskun M., Kim J. (2023). Inflammation alters iron distribution in bone and spleen in mice. Metallomics.

[54] Fisher A.L., Phillips S., Wang C.Y., Paulo J.A., Xiao X., Xu Y., Moschetta G.A., Xue Y., Mancias J.D., Babitt J.L. (2025). The hepcidin-ferroportin axis modulates liver endothelial cell BMP expression to influence iron homeostasis in mice. Blood.

[55] Hoving V., Korman S.E., Antonopoulos P., Donker A.E., Schols S.E.M., Swinkels D.W. (2022). IRIDA phenotype in TMPRSS6 monoallelic-affected patients: Toward a better understanding of the pathophysiology. Genes.

[56] Abediasl M., Siavash M., Esfahani S.S., Amini Z., Amiri K., Saket S., Esmaeili E., Rafizadeh F., Saraei R., Fakhr M.S. (2026). Iron, inflammation, and intestinal tumors: The crucial triad in colorectal cancer progression and therapy. Clin. Transl. Oncol..

[57] Varga E., Pap R., Jánosa G., Sipos K., Pandur E. (2021). Hepcidin expression via the BMP/SMAD pathway by altering BMP6, TMPRSS6 and TfR2 expressions at normal and inflammatory conditions in BV2 microglia. Neurochem. Res..

[58] Babar S., Siddiqi M.Y.J., Rizwan M., Ahmed S., Saboor M. (2026). Comparative analysis of erythroferrone and hepcidin as emerging biomarkers of iron homeostasis in patients with β-thalassemia major: A case-control study from Pakistan. Hemoglobin.

[59] Appiah S.K., Nkansah C., Appiah G.A., Abbam G., Osei-Boakye F., Daud S., Mensah K., Adwoa S., Kuwaahsuore K.S., Yeboah E. (2025). Erythroferrone-driven regulation of hepcidin and iron levels in polytransfused sickle cell anaemia patients: A prospective study. BioMed Res. Int..

[60] Corwin H.L., Napolitano L.M. (2026). Anemia of critical illness: A concise definitive review in critical care. Crit. Care Med..

[61] Luo H., Vong C.T., Tan D., Zhang J., Yu H., Yang L., Zhang C., Luo C., Zhong Z., Wang Y. (2021). Panax notoginseng Saponins Modulate the Inflammatory Response and Improve IBD-Like Symptoms via TLR/NF-κB and MAPK Signaling Pathways. Am. J. Chin. Med..

[62] Wang G., Chen Z., Song Y., Wu H., Chen M., Lai S., Wu X. (2022). Xueshuantong injection alleviates cerebral microcirculation disorder in middle cerebral artery occlusion/reperfusion rats by suppressing inflammation via JNK mediated JAK2/STAT3 and NF-κB signaling pathways. J. Ethnopharmacol..

[63] Zhang W., Zhou Y., Cui Y., Sun Y., Jiang Y., Liu J., Gong B., Wu Y., Li S., Zhuang L. (2025). Investigating the anti-atherosclerotic mechanisms of Panax notoginseng saponins through integrated cell experiments, online preparative high-performance liquid chromatography, network pharmacology, molecular docking, and molecular dynamics simulations. Biochem. Biophys. Res. Commun..

[64] Shi Y., Chen X., Luo S., Chen A., Xie K., Fang M., Zhou J. (2026). Panax notoginseng saponins ameliorate spinal cord injury by inhibiting JAK2/STAT3-mediated macrophage polarization: Enhanced delivery by ultrasound-targeted microbubble destruction. Phytomedicine.

[65] Shin Y.M., Jung H.J., Choi W.Y., Lim C.J. (2013). Antioxidative, anti-inflammatory, and matrix metalloproteinase inhibitory activities of 20(S)-ginsenoside Rg3 in cultured mammalian cell lines. Mol. Biol. Rep..

[66] Lee J.W., Choi Y.R., Mok H.J., Seong H.A., Lee D.Y., Kim G.S., Yoon J.H., Kim K.P., Kim H.D. (2017). Characterization of the changes in eicosanoid profiles of activated macrophages treated with 20(S)-ginsenoside Rg3. J. Chromatogr. B Anal. Technol. BioMed Life Sci..

[67] Tao D.J., Li R., Teng J., Yang Y., Zhang L., Zhang X., Yang R.H., Su H.Y., Shen Z.Q., Chen P. (2025). 20(R)-ginsenoside Rg3 inhibits neuroinflammation induced by cerebral ischemia/reperfusion injury by regulating the Toll-like receptor 4/myeloid differentiation factor-88/nuclear factor kappa B signaling pathway. Chem. Biodivers..

[68] Zhou Y.D., Hou J.G., Liu W., Ren S., Wang Y.P., Zhang R., Chen C., Wang Z., Li W. (2018). 20(R)-ginsenoside Rg3, a rare saponin from red ginseng, ameliorates acetaminophen-induced hepatotoxicity by suppressing PI3K/AKT pathway-mediated inflammation and apoptosis. Int. Immunopharmacol..

[69] Yu Q., Zeng K.W., Ma X.L., Jiang Y., Tu P.F., Wang X.M. (2017). Ginsenoside Rk1 suppresses pro-inflammatory responses in lipopolysaccharide-stimulated RAW264.7 cells by inhibiting the Jak2/Stat3 pathway. Chin. J. Nat. Med..

[70] Kim T.W., Joh E.H., Kim B., Kim D.H. (2012). Ginsenoside Rg5 ameliorates lung inflammation in mice by inhibiting the binding of LPS to toll-like receptor-4 on macrophages. Int. Immunopharmacol..

[71] Liu M., Ye X., Zhang S., Cheng S., Xu W., Wang X., Wen J., Li C., Yang L., Xu X. (2023). Homotherapy for heteropathy of Alzheimer’s disease and anemia through reducing the expression of toll-like receptor and TNF by steamed Panax notoginseng. BioMed Pharmacother..

[72] Zhu Z., Bai Z., Cui Y., Li X., Zhu X. (2025). The potential therapeutic effects of Panax notoginseng in osteoporosis: A comprehensive review. Phytomedicine.

[73] Liu D., Zhao Z., Jiang W., Zhu P., An X., Xie Y., Huang X., Zhou N. (2021). Panax notoginseng saponin promotes bone regeneration in distraction osteogenesis via the TGF-β1 signaling pathway. Evid. Based Complement. Altern. Med..

[74] Wrighting D.M., Andrews N.C. (2006). Interleukin-6 induces hepcidin expression through STAT3. Blood.

[75] Verga Falzacappa M.V., Casanovas G., Hentze M.W., Muckenthaler M.U. (2008). A bone morphogenetic protein (BMP)-responsive element in the hepcidin promoter controls HFE2-mediated hepatic hepcidin expression and its response to IL-6 in cultured cells. J. Mol. Med..

[76] Casanovas G., Banerji A., D’Alessio F., Muckenthaler M.U., Legewie S. (2014). A multi-scale model of hepcidin promoter regulation reveals factors controlling systemic iron homeostasis. PLoS Comput. Biol..

[77] Canali S., Core A.B., Zumbrennen-Bullough K.B., Merkulova M., Wang C.Y., Schneyer A.L., Pietrangelo A., Babitt J.L. (2016). Activin B induces noncanonical SMAD1/5/8 signaling via BMP type I receptors in hepatocytes: Evidence for a role in hepcidin induction by inflammation in male mice. Endocrinology.

[78] Meynard D., Kautz L., Darnaud V., Canonne-Hergaux F., Coppin H., Roth M.P. (2009). Lack of the bone morphogenetic protein BMP6 induces massive iron overload. Nat. Genet..

[79] Won H.J., Kim H.I., Park T., Kim H., Jo K., Jeon H., Ha S.J., Hyun J.M., Jeong A., Kim J.S. (2019). Non-clinical pharmacokinetic behavior of ginsenosides. J. Ginseng Res..

[80] Hu Q.R., Hong H., Zhang Z.H., Feng H., Luo T., Li J., Deng Z.Y., Chen F. (2023). Methods on improvements of the poor oral bioavailability of ginsenosides: Pre-processing, structural modification, drug combination, and micro- or nano-delivery system. J. Ginseng Res..

[81] Yao Z.W., Zhu H. (2025). Pharmacological mechanisms and drug delivery systems of ginsenoside Rg3: A comprehensive review. Pharmacol. Res..

[82] Kim S.W., Han B.C., So S.H., Han C.K., In G., Park C.K., Hyun S.H. (2023). Biodistribution and pharmacokinetic evaluation of Korean Red Ginseng components using radioisotopes in a rat model. J. Ginseng Res..

[83] Bloomer S.A., Brown K.E. (2021). Hepcidin and iron metabolism in experimental liver injury. Am. J. Pathol..

[84] Wu W., Song Y., He C., Liu C., Wu R., Fang L., Cong Y., Miao Y., Liu Z. (2015). Divalent metal-ion transporter 1 is decreased in intestinal epithelial cells and contributes to the anemia in inflammatory bowel disease. Sci. Rep..

[85] Shi Y., Chen J., Qu D., Sun Q., Yu Y., Zhang H., Liu Z., Sha J., Sun Y. (2024). Ginsenoside Rg5 activates the LKB1/AMPK/mTOR signaling pathway and modifies the gut microbiota to alleviate nonalcoholic fatty liver disease induced by a high-fat diet. Nutrients.

[86] Wei Y., Yang H., Zhu C., Deng J., Fan D. (2020). Hypoglycemic effect of ginsenoside Rg5 mediated partly by modulating gut microbiota dysbiosis in diabetic db/db mice. J. Agric. Food Chem..

[87] Chen Y., Zhang K.X., Liu H., Zhu Y., Bu Q.Y., Song S.X., Li Y.C., Zou H., You X.Y., Zhao G.P. (2024). Impact of ginsenoside Rb1 on gut microbiome and associated changes in pharmacokinetics in rats. Sci. Rep..

[88] Zhou C., Zhu M., Li Q., Deng W., Sun N., Liu W., Li Y., Wang X., Zhang F., Li L. (2026). Functional characterization of a novel β-glucosidase converting trillin to diosgenin from Enterococcus faecalis. Microb. Cell Factories.

[89] Jiang M., Zhu L., Xie S., Ren Z., Chen X., Liu M., Yin G. (2024). Transcriptome profiling, cloning, and characterization of AnGlu04478, a ginsenoside-hydrolyzing β-glucosidase from Aspergillus niger NG1306. Curr. Microbiol..

[90] Li Q., Wang L., Fang X., Zhao L. (2022). Highly Efficient Biotransformation of Notoginsenoside R1 into Ginsenoside Rg1 by Dictyoglomus thermophilum β-xylosidase Xln-DT. J. Microbiol. Biotechnol..

[91] Diao M., Chen Y., Meng L., Li J., Xie N. (2024). Biotransformation approach to produce rare ginsenosides F1, compound Mc1, and Rd2 from major ginsenosides. Arch. Microbiol..

[92] Qu Q., Zhou Q., Peng X., Li Z., Jia M., Zhang Y., Yang C., Dai X., Sheng M., Kang J. (2026). Monascus fermentation enhances the lipid-lowering properties of ginseng in vivo and component assay. Food Res. Int..

[93] Wei W., Li M., Pan L., Shao M., He X., Li Y., Sheng L., Zheng N., Li H. (2025). Inter-species and individualized biotransformation of five saponins by human being- and mouse-derived fecal microbiota. Chin. Med..

[94] El-Haddad A.E., Abd El-Al M.S., Teleb M., Farag M.A. (2026). Antidiabetic action mechanisms of saponins via gut digestive enzymes inhibition and microbiota interaction, a comprehensive review and in silico docking to improve efficacy. Eur. J. Med. Chem..

[95] Zhai S., Peng X., Liu C., Zhang R., Jin C., Jiang X., Feng P., Liang Y., Yuan X., Zhang J. (2025). Ginsenoside Rg1 alleviates ochratoxin A-induced liver inflammation in ducklings: Involvement of intestinal microbiota modulation and the TLR4/NF-κB pathway inhibition. Ecotoxicol. Environ. Saf..

[96] Wang L., Shao L., Chen M.Y., Wang L., Zhang W., Tan F.B., Huang W.H. (2022). Effect of ginsenoside compound K on alleviating colitis via modulating gut microbiota. Chin. Med..

